# Stimulatory Effect of Xenobiotics on Oxidative Electron Transport of Chemolithotrophic Nitrifying Bacteria Used as Biosensing Element

**DOI:** 10.1371/journal.pone.0053484

**Published:** 2013-01-09

**Authors:** Andrzej Woznica, Agnieszka Nowak, Przemyslaw Ziemski, Mirosław Kwasniewski, Tytus Bernas

**Affiliations:** 1 Department of Biochemistry, Faculty of Biology and Environmental Protection, University of Silesia, Katowice, Poland; 2 Department of Genetics, Faculty of Biology and Environmental Protection, University of Silesia, Katowice, Poland; 3 Nencki Institute of Experimental Biology, Polish Academy of Sciences, Warszawa, Poland; Institut Pasteur Paris, France

## Abstract

Electron transport chain (ETCh) of ammonium (AOB) and nitrite oxidizing bacteria (NOB) participates in oxidation of ammonium to nitrate (nitrification). Operation of ETCh may be perturbed by a range of water-soluble xenobiotics. Therefore, consortia of nitrifying bacteria may be used as a biosensor to detect water contamination. A surprising feature of this system is an increase of oxygen consumption, detected in the presence of certain inhibitors of ETCh. Thus, to shed light on the mechanism of this effect (and other differences between inhibitors) we monitored separately respiration of the bacteria of the first (AOB - *Nitrosomonas*) and second (NOB -*Nitrobacter*) stages of nitrification. Furthermore, we measured plasma membrane potential and the level of reduction of NAD(P)H. We propose a novel model of ETCh in NOB to explain the role of reverse electron transport in the stimulation of oxygen consumption (previously attributed to hormesis).

## Introduction

Nitrification is a biological oxidation of ammonium to nitrate, with nitrite as the intermediate product. The two stages of this reaction are catalyzed by two groups of ubiquitous lithoautotrophic bacteria. The first comprises ammonium-oxidizing bacteria (AOB), whereas the second nitrite-oxidizing bacteria (NOB) [1]–[8]. The respective reactions, catalyzed by ammonia monooxygenase (AMO, in AOB) or nitrite reductase (NXR, in NOB), constitute elements of electron transport chain (ETCh). Operation of ETCh may be perturbed by a wide range of water-soluble xenobiotics. For instance, NADH/ubiquinone (UQ) oxidoreductase can be inhibited by rotenone, capsaicin and dicumarol [9]–[11] and flavin oxidoreductase by quinacrine [12]. The latter, being an isoalloxazine analog, may also act as a competitive inhibitor of molybdopteridin cofactors [13]. Another group comprises inhibitors of cytochrome c oxidase aa3: cyanide, azide and carbon monoxide [14]. Moreover, other xenobiotics (ex. phenol) may inhibit cellular respiration in an indirect fashion, without involvement of ETCh [8], [15]. Hence, consortia of nitrifying bacteria can be used as biosensing element to detect diverse forms of water contamination [8], [16]. One should note that AMO and NXR are unique to nitrifying bacteria (AOB and NOB, respectively). Furthermore, it has been postulated that ETCh of NOB is shorter than its AOB counterpart. The NOB ETCh includes UQH_2_/cytochrome c oxidoreductase and cytochrome aa3 oxidase, functionally similar to mitochondrial complex III and IV, respectively [12], [17], [18]. This notion is in accordance with low oxidation/reduction potential of the NO_2_^−/^NO_3_^−^ pair [12], which makes concomitant reduction of quinone thermodynamically unfavorable. Moreover, UQH_2_/cytochrome c and NADH/UQ oxidoreductases (functionally similar to complexes III and I in mitochondria, respectively) of the NOB ETCh were reported to catalyze together (reversed) transport of electrons from NO_2_^−/^NO_3_^−^ pair to NAD(P)/NAD(P)H pair [12]. This endergonic reaction utilizes the electric potential across plasma membrane of NOB [12], [17], [18], [19]. Increase of oxygen consumption in consortia of nitrifying bacteria, detected in the presence of some inhibitors of mitochondrial ETCh, is another surprising feature of this system [8]. This behaviour (hormesis), attributed to overcompensation of the response aimed at restoring homeostasis [20], [21], is not unique to bacteria [22]. However, mechanism of this effect has not been established. The aim of this study was therefore to elucidate involvement of ETCh in putative hormesis and thereby facilitate design and operation of biosensors based on nitrifying bacteria. As demonstrated previously, stable operation of such biosensors requires mature consortia characterized by stable metabolism, population composition and spatial architecture [7]. It should also be noted that biosensing is performed using consortia in equilibrium with flowing water [7].

Therefore, in this study, oxygen consumption of such consortia is measured using electron donors which are specific for either AOB or NOB. Moreover, population composition is characterized in details (with genomic analysis) to validate the biochemical results and to assist in their generalization.

## Materials and Methods

### Bacterial Culture

Bacteria were isolated from activated sludge from water treatment plant Klimzowiec, Katowice, Silesia [23]. Consortia of nitrifying bacteria were grown in 10 L laboratory scale reactor (Braun Melsulgen Biostat), using mineral liquid medium (MLM) [24]. The reactor was aerated continuously at 2 L min^−1^ rate under conditions of continuous mixing (500 RPM). Oxygen concentration was approximately 5 mg L^−1^, pH was 7.5, and temperature was 20°C. Bacteria were harvested with centrifugation (9000 rpm, 50 min) of 1.5 L of the bacterial culture. The pellet was re-suspended in 70 mL of MLM without ammonium and nitrite. Ammonium and/or nitrite were added directly at the onset of measurements. Dry bacterial biomass was estimated with use standard weight method [25]. During batch experiments bacterial activity was converted to bacterial biomass using calibration curve of activity versus the biomass. All necessary permits were obtained for the described field Chorzowsko- Świętochłowickie Przedsiębiorstwo Wodociągów i Kanalizacji sp. z o.o as the owner of water treatment plant Klimzowiec in Katowice, Silesia [23].

### Metagenomic Analysis of Bacterial Community Composition

200 mL water samples were filtered through 0.45 µm pore sized filters (Sartorius) to collect total microbial community. Directly after filtration, total DNA was extracted from filters with the PowerWater DNA Isolation Kit (MO BIO Laboratories, Carlsbad, CA) following the manufacturer’s instructions. The V1–V3 regions of the bacterial 16S rRNA gene were amplified in PCR with the following primer set, containing at the 5′ ends sequences of A and B sequencing primers (454/Roche; underlined): BSF8: 5′-CGTATCGCCTCCCTCGCGCCATCAGAGTTTGATCCTGGCTCAG-3′ and the USR515: 5′- CTATGCGCCTTGCCAGCCCGCTCAGCACCGCGGCKGCTGGCAC-3′ [26]. PCR were prepared in 20 µL reactions contained 1×Phusion HF Buffer (Thermo Scientific, Wilmington, USA), 0.4 U Phusion High-Fidelity DNA Polymerase (Thermo Scientific, Wilmington, USA), 0.2 mM deoxyribonucleoside triphosphates, 0.1 µM each of forward and reverse primers and 20 ng of DNA template. PCR was performed in a thermal cycler TProfessional (Biometra, Goettingen, Germany) under the following condition: initial denaturation at 98°C for 30 s; 35 cycles at 98°C for 10 s, 56°C for 15 s, and 72°C for 10 s; and a final extension at 72°C for 5 min. Amplicons were purified using the AgencourtAMPure XP (Beckman Coulter Inc, Mississauga, USA), and quantified using the Quant-iT™ PicoGreen® dsDNA Assay kit (Invitrogen, Burlington, USA) using the TBS-380 Fluorometer (Turner Biosystems, CA, USA) following the “Amplicon Library Preparation Method Manual” of the 454 GS Junior Titanium System (454 Life Sciences/Roche, Branford, USA). Emulsion PCR was performed according to the “em-PCR Amplification Method Manual –Lib A” and sequencing was performed in a single run of a 454 GS Junior Titanium System following the “Sequencing Method Manual” (454 Life Sciences/Roche).

Sequences were processed and analyzed using the Quantitative Insights Into Microbial Ecology pipeline (QIIME v1.5.0; [27]; http://qiime.org/) with standard settings. First, all reads from the original 454 FASTA file (*.fna file) were screened for sequences containing the reverse primer sequence, and these were used in subsequent steps of the analysis. After trimming of primer sequences, processed sequences were clustered based on their sequence similarity into Operational Taxonomic Units (OTUs) at 97% pairwise identity. Representative sequences from each OTU were selected automatically and aligned to the Greengenes imputed core reference alignment (Greengenes version 12_10; [28]; http://greengenes.lbl.gov). Chimeras were removed using Chimera Slayer [29]. Taxonomy assignments were prepared using the Naïve Bayesian rRNA Classifier Version 2.5 [30] of the Ribosomal Database Project (RDP; [31]; http://rdp.cme.msu.edu/).

### Determination of Bacteria Metabolic Activity in Bulk

The velocity of forward electron transport (the main component of nitrification) was monitored using consumption of oxygen (the electron acceptor). This parameter was measured in a mixture of 4 mL of bacteria suspension and 6 mL of aerated MLM enriched with ammonium (initial conc. 4 mM) or nitrite (initial conc. 1.5 mM). These specific electron donors permitted independent assessment of AOB and NOB, respectively. Measurements were performed in 20 mL polystyrene closed chamber placed on a magnetic stirrer (240 rpm). Oxygen level was monitored with galvanic electrodes (Senco CTN-9202 S). The measurements (minimal accuracy 0.1 mg L^−1^) were taken for 6 minute at 10 s intervals, using a digital transducer [23].

The kinetics of nitrification was characterized according to Michaelis-Menten model, with the parameters (Km and Vmax) estimated at the optimal conditions (pH 7.5, temp 22°C), as described earlier [7]. The model parameters were determined in the ranges of concentrations between 0–8 mM of ammonia and 0–30 mM of nitrite were examined [7]. Effects of inhibitors were studied out at the optimum (Km) concentrations of ammonium (7 mM, AOB) or nitrite (15 mM, NOB). The following inhibitors were used: cyanide (0.25–5.50 µM), azide (0.5–3.0 mM), quinacrine (0.05–1.00 mM) and dicumarol (40–160 µM). Stock solutions of these compounds were prepared in water, with the exception of dicumarol which was dissolved in the 0.5 M NaOH. All results were normalized to the respective control measured in the absence of inhibitors.

The amount of reduced pyridine nucleotides was monitored with intrinsic fluorescence of their oxidized forms [32], [33]. This autofluorescence of NAD(P)H was excited at 360 nm and detected at 450 nm using Hitachi 7000 FL spectrofluorimeter. The excitation and emission slits were set to 5.0 nm whereas the PMT gain was set to 250 V. One should note that the method did not differentiate between NADPH and NADH. Thus, although the bacterial UQ oxidoreductase is expected to predominantly react with NADH/NADP^+^ pair, we use the term NAD(P)H in this text.

### Labeling and Imaging of Bacteria

The AOB and the NOB were localized in the bacterial consortia by fluorescence *in situ* hybridization using commercially available Nitri-VIT kits (vermicon AG, Germany). Nitri-VIT contains a mixture of oligonucleotide DNA probes complementary to specific 16S rRNA regions of described AOB from environmental fresh water samples (detected genera: *Nitrosomonas, Nitrosococcus, Nitrosospira*) and NOB (detected genera: *Nitrobacter, Nitrospira*) [7]. The probe sequence specificities were validated *in silico* using the function “probe match” of the software package ARB [34].

In order to measure plasma membrane potential 400 µL of biomass was suspended in medium containing NO_2_^−/^NH_4_^+^ and JC-1 (8 µM). The incubation was performed for 30 min. in a chamber made of 1 mL eppendorf with trimmed tip, glued to the coverslip. The chamber was used to image the stained bacteria with a Olympus FV1000 (Olympus, Poland) confocal system equipped with a Olympus IX81 inverted microscope 60×PlanApo water immersion objective lens (NA 1.2,) and a 100 mW multiline argon ion laser (MellesGriot BV, The Netherlands). The system was equipped with 405/488 nm 488/543/633 nm double primary dichroic mirrors and a 560 nm secondary dichroic mirror. Following the FISH labeling, fluorescence of NOB was detected in the 500–550 nm emission range (488 nm excitation, whereas the AOB fluorescence in the 560–660 nm range (543 nm excitation), collected sequentially (to prevent crosstalk). Fluorescence of monomeric (green) and aggregate (red) forms of JC-1 was excited with 488 nm light. The respective detection bands were 500–550 nm (monomer) and 560–630 nm (aggregate). Single optical sections through the bacterial consortia (approx. 900 thickness) were registered with the confocal pinhole set to 1 Airy unit using two separate photomultipliers (R6357, Hamamatsu, Japan) working in integration mode at 12-bit precision of signal digitization (4096 intensity levels). The membrane potential was estimated using red/green (JC-1 aggregate/monomer) fluorescence intensity ratio calculated on pixel-by-pixel basis. Change of the potential was monitored by comparison of the ratio scatterplots corresponding to the consortia sample before and after treatment with inhibitors.

### Statistics

Bacterial biomass was estimated from the activity (measured in standard conditions) using a calibration curve calculated using least-squares regression (MS Excel).

Velocity of nitrification was defined as first derivative (extrapolated to 0) of the magnitude of oxygen consumption as a function of time (10 s time interval, 45 measurements). On the basis of that data half-saturation constants Km_(AOB)_, Km_(NOB)_ and nitrification velocities V_AOB_, V_NOB_ were determined using iteration method with Gauss-Newton algorithm (Statistica 8.0 Statsoft, US). One should note that the contribution of secondary oxidation of nitrite formed from ammonium was eliminated from measurements of AOB activity. Statistical comparisons were executed with analysis of variance (ANOVA), under MS Excell.

## Results

### Composition and Activity of Unperturbed Consortia of Nitrifying Bacteria

**Figure 1:**
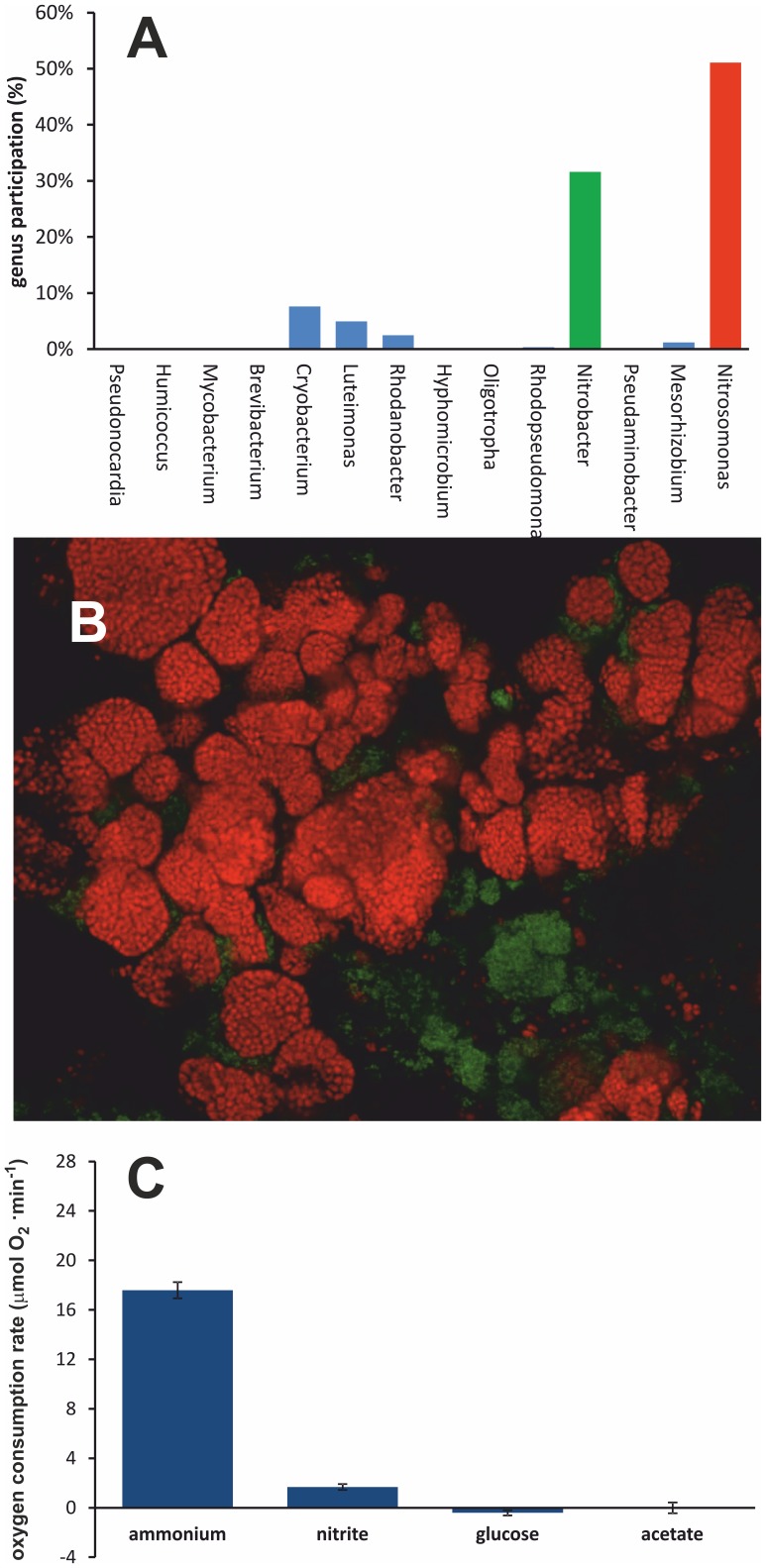
Generic composition, spatial architecture and metabolic activity of studied microorganisms consortia. A – Population composition of bacterial consortia (measured with relative abundancy of specific 16S rDNA genes). B **–** Microscope image of consortia of nitrifying bacteria: AOB (red) and NOB (green), labeled using FISH. C – Metabolic activity of the consortia measured in the presence of ammonium, nitrite, glucose and acetate. Biomass 0.675 mg mL^−1^, concentration of ammonium 4 mM, nitrite 1.5 mM, glucose 2.8 mM, acetate 8.3 mM, temp 20±1°C, pH 7.5±0.1.

In our work we used the heterogenous consortia of AOB/NOB. Metagenomic analysis of consortia in bulk demonstrated that the consortia comprised mainly (82.7%) nitrifying bacteria (AOB and NOB). The former belonged to the *Nitrosomonas* genus (51.1%), whereas the latter to the *Nitrobacter* (31.5%) and *Nitrococcus* (0.02%) genera (Fig. 1A). The remaining 17.3% were other bacteria, which do not participate in the nitrification process (Fig. 1A). The presence and localization of AOB and NOB in the consortia was further ascertained with FISH labeling, followed by imaging with confocal microscopy (Fig. 1B). Metabolic activity of bacterial consortia of nitrifying bacteria was measured in the presence of several organic compounds (potential carbon sources). In the medium containing glucose or acetate (organic compounds) observed oxygen consumption was negligible and not different from the control (Fig. 1C). However, elevated rates of oxygen consumption in the initial inoculum were detected in the presence of 7 mM ammonium or 15 mM nitrite (Fig. 1C). These observations indicated that examined process had lithoauthotrophic character, and was catalyzed by consortia of AOB and NOB. Other bacteria, although present, were unlikely to influence the measured activity of the consortium. Moreover, the activity of AOB in the inoculums was 3 times higher than NOB. This notion was compatible with the complete ammonium oxidation process stoichiometry. One may note that the activity in the presence of optimal carbon source (NaHCO_3_) was proportional to the dry mass of the consortia sample.

### Change of Activity of Consortia of Nitrifying Bacteria in the Presence of ETCh Inhibitors

#### Inhibiton of aa3 oxidase

**Figure 2:**
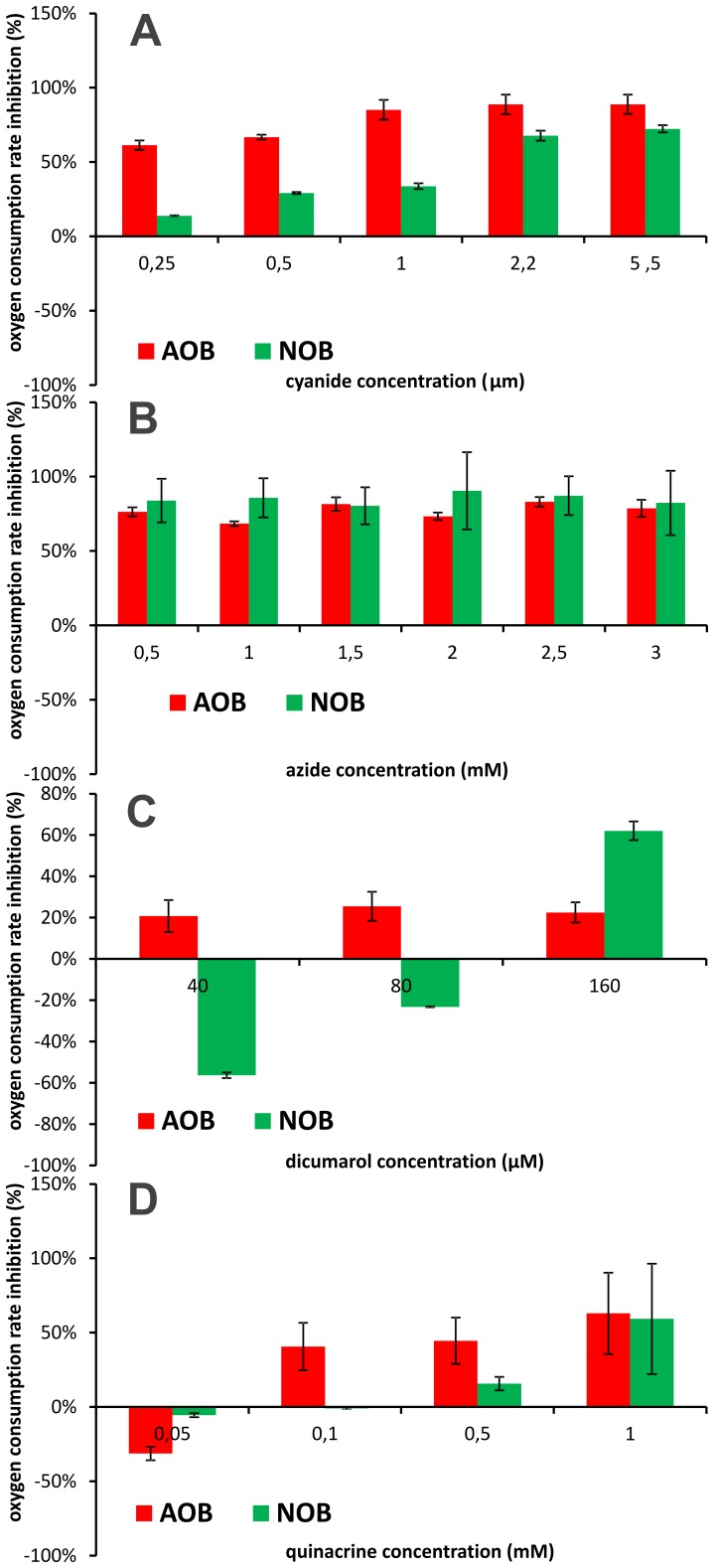
Change of oxygen consumption rate in the presence of cyanide (A), azide (B), dicumarol (C) and quinacrine (D). Ammonium concentration 4 mM (AOB), nitrite concentration 1.5 mM (NOB), temp 20±1°C, pH 7.5±0.1.

Azide and cyanide were used to block cytochrome aa3 oxidase, as described in [12], [15]. In the presence of these inhibitors a decrease of oxygen consumption in both bacterial groups (AOB and NOB) was observed (Fig. 2A and 2B). One may note that the magnitude of inhibition increased with concentration of cyanide but was not affected by concentration of azide. Furthermore, in the presence of cyanide, the relative decrease in activity of NOB was higher than that of AOB.

#### Inhibition of UQH_2_/cytochrome c oxidoreductase

Dicumarol and quinacrine were used inhibit UQH_2_/cytochrome c oxidoreductase [23], [34]. Inhibition of oxygen consumption in AOB was observed in the whole range of concentrations of the former agent. The magnitude of this effect increased only slightly with concentration of dicumarol (Fig. 2C). However, the stimulation of NOB activity was detectable at low concentrations of dicumarol (40 and 80 µM), whereas at higher concentrations (160 µM) the activity was inhibited (Fig. 2C). Different pattern of inhibition was observed in the presence of quinacrine (Fig. 2D). Significant decrease of the oxygen consumption was detectable only above certain concentrations of this xenobiotic (Fig. 2D). Below these thresholds quinacrine caused an increase in the oxygen consumption of both AOB and NOB (Fig. 2D). It should be noted that maximum decrease in oxygen consumption was higher in AOB (65%) than in NOB (49%).

#### Effect of inhibitors on the redox state of reduction of pyridine nucleotides

**Figure 3:**
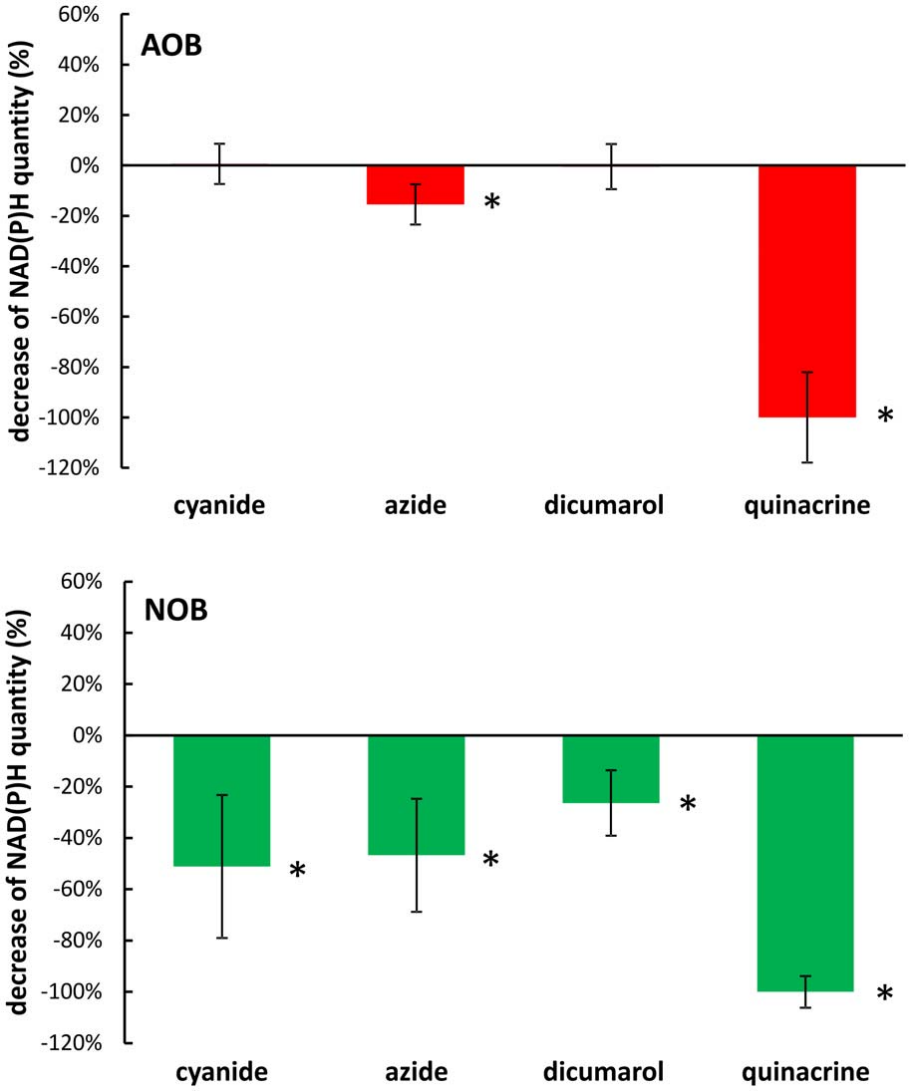
Relative decrease of NAD(P)H (with respect to the control) in AOB and NOB cells in the presence of cyanide (2.0 µM), azide (1.5 mM), dicumarol (80 µM) and quinacrine (0.5 mM), relative to the control without inhibitors. Statistically significant differences are indicated with asterisk. Ammonium concentration (AOB) 4 mM, nitrite concentration (NOB) 1.5 mM, temp 20±1°C, pH 7.5±0.1.

Redox state of NAD(P)H was estimated (using the fluorescence of its reduced form) in the bulk of consortia sample, in the presence of the four inhibitors (cyanide, azide, quinacrine and dicumarol). No significant change was detectable when AOB were studied in the presence of cyanide, azide and dicumarol (Fig. 3A). However, nearly complete oxidation NAD(P)H in the was detected in the presence of quinacrine (Fig. 3A). Similar effect occurred when NOB were activated. However, in that case significant decrease of NAD(P)H concentration was observable also in case of the other three inhibitors (cyanide, azide and dicumarol) although the oxidation of the nucleotide was less pronounced than in the case of quinacrine (Fig. 3B).

#### Effect of inhibitors on plasma membrane potential

The plasma membrane potential was estimated with a fluorescent cation which accumulates inside cells according to Nernst equilibrium and forms aggregates (red fluorescence) at high concentration whereas its monomeric form (green fluorescence) dominates at low concentration.

**Figure 4:**
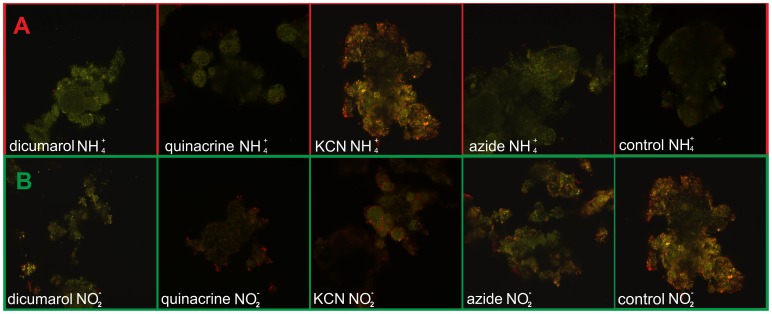
Plasma membrane potential of AOB (row A), NOB (row B), visualized by JC-1 (8 µM) in the presence of cyanide (100 **µM), azide (75 mM), dicumarol (25 µM), quinacrine (1 mM) and control without xenobiotics.** Observation started after 30 min. preincubation in the eppendorf with coverslip. Ammonium concentration 4 mM, nitrite concentration 1.5 mM, temp 20±1°C, pH 7.5±0.1.

Only minor changes in the JC1 red/green ratio were observed both in AOB and NOB in the presence of azide in the opposite to the control (Fig. 4A and 4B). This compound induced slight loss of plasma membrane potential (depolarization) in majority of AOB and some NOB cells. On the other hand a slight increase of the potential was detected in NOB, but not AOB in the presence of dicumarol (Fig. 4A and 4B). Surprisingly, however, a marked increase of JC1 red/green ratio was observable in NOB treated with cyanide (Fig. 4A and 4B). Similar, but stronger effect occurred in both AOB and NOB treated with quinacrine (Fig. 4A and 4B).

## Discussion

The purpose of this study was to elucidate the stimulatory effect of inhibitors of ETCh on oxygen consumption in consortia of nitrifying bacteria used as a bioelement in sensing of xenobiotics [8]. Even though these consortia were derived from waste water [23] their population composition comprised only a minor fraction of bacteria non-related to nitrification (as compared to AOB and NOB). It may also be noted that the presented data are in accordance with our earlier observations, which indicated that the AOB constituted the majority of bacterial population of the consortia [7]. Moreover, as electron donors specific for AOB and NOB were used exclusively, the activity of other bacteria in our system was negligible [7], [8]. Therefore, the presence of bacteria which did not belong to either AOB or NOB was unlikely to affect any of the parameters measured in this study: oxygen consumption, membrane potential and redox state of NAD(P)H.

A decrease of oxygen consumption was observed in AOB and NOB, in the presence of both azide and cyanide. These substances produced a plateau of inhibition magnitude. This pattern is in accordance with the mechanism of action of both substances which are non-competitive inhibitors of heme–copper cytochrome aa3 oxidase superfamily. It should be noted that enzymes of this class are structurally similar in both groups of bacteria [35]–[38]. Moreover, lack of increase of reduced NAD(P)H pool indicates that inhibition of cytochrome aa3 does not divert electrons to the reversed transport pathway of NOB [12]. Instead a minor oxidation of the nucleotides was detected. Furthermore, the absence of NAD(P)H oxidation in AOB suggests that cytochrome aa3 is not involved in reverse electron transport in these bacteria. It should be noted that the enzyme (UQ oxidoreductase) catalyzing the last step of this process is expected to react with NADH/NADP^+^ pair. However, as our method did not differentiate between NADPH and NADH we use the term NAD(P)H in this text. One may note that cyanide, but not azide produces an increase of membrane potential, which is not compatible with inhibition of proton pump coupled to the operation of cytochrome aa3 oxidase. However, it is possible that the electric potential of the bacterial membrane is generated not only by protons also by other cations. For instance, concentration of K^+^ ions inside bacterial cells exceeds that of the environment [39]. Several forms of potassium channels have been detected in many groups of bacteria [39]–[42], including NOB. Activity of these channels may be modulated in the presence of CN which causes their opening [39], [43] and efflux of K^+^ to the environment making the cytoplasm more negative, as compared to the environment. Such increase of the potential in the presence of cyanide was reported in other experimental systems [44], including bacterial [40], [45]. One should also note that efflux of potassium would facilitate entry cationic probe of electric potential, JC-1 (used in this study).

Similar increase of membrane potential was also observed in the presence of quinacrine. This effect was accompanied by a slight increase (at low concentrations) of oxygen consumption of AOB and NOB and a complete oxidation of NAD(P)H pool. It should be noted that quinacrine may be reduced by NAD(P)H and transfer electrons to oxygen [46], in the process of redox cycling. This reaction may, in turn contribute to consumption of protons and oxygen inside bacterial cells (via dismutation of superoxide anion) and therefore increase the proton gradient across the plasma membrane. However, quinacrine may also block molybdopterin (isoalloxazine) from NXR [13], [47] and thus impede oxygen consumption in nitrite oxidation. This effect may predominate at high concentrations of this xenobiotic. One may hypothesize that similar mechanism exists in the case of AMO in AOB.

An increase of oxygen consumption was also detected in NOB at low concentrations of dicumarol. This stimulatory effect was not detectable in AOB, although the inhibitor was effective only above threshold concentration. Moreover, a decrease of the concentration of reduced form of NAD(P)^+^ was observed in NOB but not in AOB. These data are in agreement with reversed electron transfer, which was postulated to operate in *Nitrobacter* [12]. The electrons are introduced to the respiratory chain by NXR at cytochrome c1 level. Hence, they may be transported to oxygen (cytochrome oxidase) or to NAD(P)^+^ by UQH_2_/cyt c1 oxidoreductase and NADH/UQ oxidoreductase. The energy needed to reverse electron transport (catalyzed by these enzymes) is supplied by the proton gradient [12]. It should be noted that a minor fraction of electron transported by ETCh followed this pathway [12]. Moreover, velocity of forward electron transport is affected by the magnitude of the gradient in mitochondria and by general redox state of the cell. One should note that the Em7 for the cytochrome is +270 mV, or 150 mV more electronegative than for the pair NO_2_/NO^−^. Thus, to overcome this energy barrier oxidation of nitrite requires electric potential across the cell membrane (as an energy source) to proceed [12], [17]–[19]. Consequently, one may expect that when the dissipation of pH gradient and production of NAD(P)H (due to reversed electron transport) is completely blocked (at high concentration of inhibitor) the forward transport (generation of proton gradient) may be also inhibited (in a indirect way) by a change in cellular redox state. One may note that the postulated mechanism is compatible with increase of membrane potential, which was induced by treatment with dicumarol.

**Figure 5:**
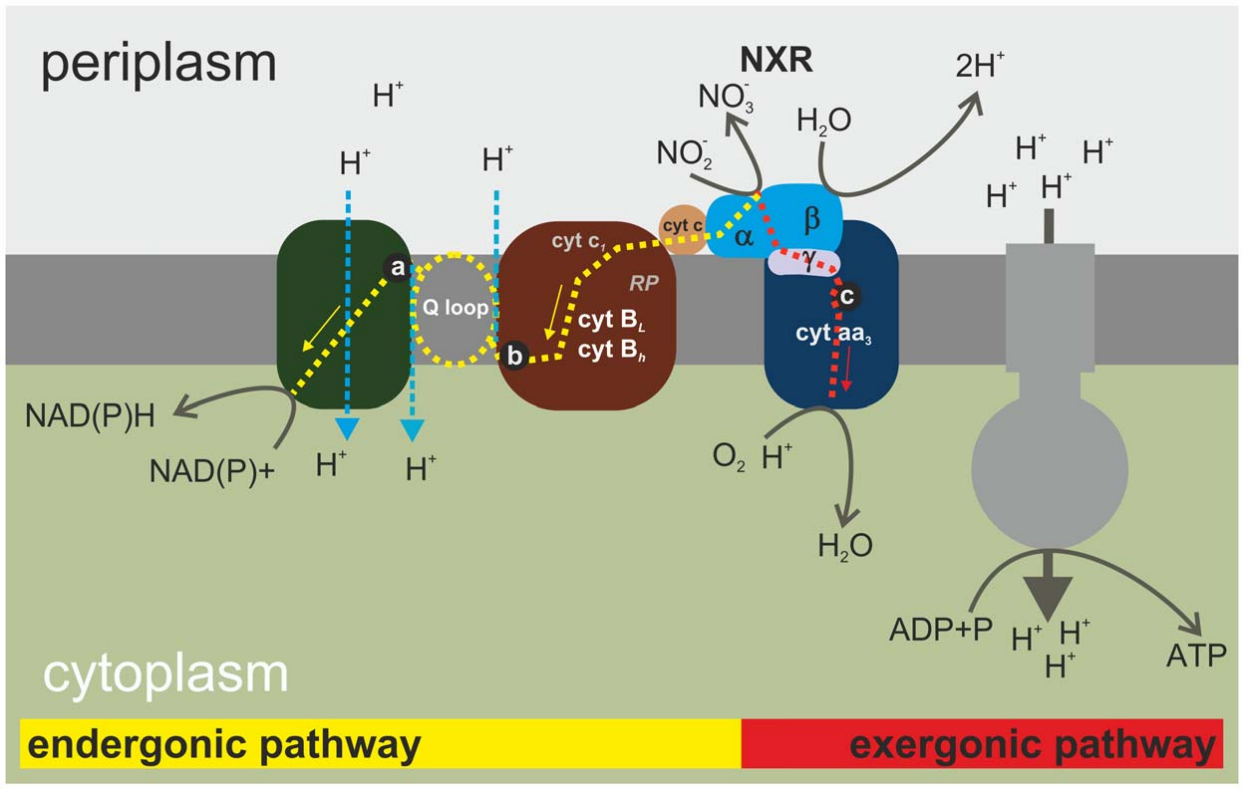
Hypothetical schematic of NOB ETCh with marked places of electron flow inhibition where a - quinacrine; b - dicumarol; c - azide and cyanide. Red arrow shown electron flow from NXR (nitrite oxidase) to oxygen; yellow arrow - reverse electron flow from NXR to NAD(P)^+^; blue arrow- reverse proton flow. The model was based on the data presented in [12], [17], [18], [47]–[51].

Taking into account the above evidence and the electron microscopy data [37]–[39] one may propose hypothetical schematics of NOB ETCh (Fig. 5). It should be noted that, in contrast to earlier models [12] the NXR acts on the outside of the plasma membrane, directly contributing to postulated by Spieck and coworkers [48]–[51] mechanism of proton gradient generation. Moreover, reduction of both NAD(P)H (NADH/UQ oxidoreductase) and oxygen (cytochrome c oxidase aa3) participate in this process by consumption of the protons on the opposite side of the plasma membrane. This model does not require proton pumping by cytochrome c oxidase aa3. We have included the reduction of NADH (catalyzed by NADH/UQ oxidoreductase). This reaction utilizes the electric potential across plasma membrane of NOB. One should also note that similar pathway has been described in ammonium-oxidizing bacteria (AOB) [12], [17]–[19], [51]–[53].

We present evidence of both stimulation and inhibition of oxygen consumption of the consortium (biofilm) of two kinds of nitrifying bacteria, described previously [7], [8]. This effect may be attributed to the presence of alternative routes of electron transport in these organisms. Moreover, this notion may form the basis of explanation of hormesis effect, reported in earlier studies. The differences between electron transport chains of AOB and NOB contribute to the kinetics and magnitude of their reactions to xenobiotics. Our data indicated that using consortium of bacteria in biosensing systems permit to detect broader range of harmful or toxic substances, compared to a sensor based on a single bacterial strain. Therefore, they constitute therefore an advantage for biosensing, facilitating detection of varied xenobiotics of varied types and concentrations. Characterization of the kinetics and mechanism of sensor response to xenobiotics is a prerequisite for proper interpretation of such data.
